# Clinical and radiographic outcomes of endocrowns fabricated from two different CAD-CAM materials versus stainless steel crowns in restoring first permanent molars in children: a randomized clinical trial

**DOI:** 10.1186/s12903-025-06507-z

**Published:** 2025-07-08

**Authors:** Ahmed Ismail Taha, Aya Ehab Saad

**Affiliations:** 1 Prosthodontic Department, Faculty of Dentistry, Kafrelsheikh University, Mubark Road, 33511 Kafr Abu Tabl, Kafrelsheikh Governorate, Kafrelsheikh, 6860404 Egypt; 2 Pediatric Dentistry and Public Health Department, Faculty of Dentistry, Delta University for science and technology, Mansoura, Egypt

**Keywords:** Crown, Root canal therapy, Permanent molars, Plaque index and pediateric dentistry

## Abstract

**Background:**

Restoring first permanent molars after endodontic treatment in children is challenging. Improved mechanical properties and adhesion of ceramic materials have led to the emergence of endocrown as a conservative and esthetic restorative option for endodontically treated molars in adults and offer dentists a restorative treatment for endodontically treated first permanent molars in children. The purpose of this study was clinical and radiographic evaluation of both endocrowns fabricated from 2 different materials and SSCs restoring endodontically treated first permenant molars in children over one year.

**Methods:**

Thirty children were selected (18 girls and 12 boys) with an age range of 10–13 years old, with an endodontically treated first molar. Children were randomly divided into 3 groups: PMC group (restored with preformed SSCs), EMX group (restored with litium disilicate endocrown), and COP group (restored with indirect reinforced composite endocrown) (*n* = 10). Evaluation was done in terms of parent satisfaction, radiograph (base line and 12 months), the restoration survival after 12 months, plaque index (PI), and gingival index (GI) at base line, 6 months, and 12 months.The data were analyzed using the Kruskal-Wallis H-test, which was used to compare an ordinal variable, and Friedman’s test was used to compare an ordinal variable (*P* ≤.050).

**Results:**

Parent satisfaction showed statistically significant differences between PMC (mean rank = 7.5) and both EMX and COP (mean rank = 19.5) (*P* <.001), but not between the EMX and COP groups (*P* = 1.00). At 6 and 12 months, the PMC group’s PI values were statistically significantly higher than those of the EMX and COP groups (*P* =.001 and *P* <.001, respectively). The GI values of the EMX and COP groups did not change significantly through different intervals (*P* = 1.000 and *P* =.135, respectively), whereas the GI values of the PMC group did (*P* =.050). At various intervals, it was found that there was no significant difference in the GI values between the three groups.

**Conclusions:**

The survival rates of endorowns and SSCs were comparable. Compared to SSCs, endocrowns demonstrated a higher parental satisfaction, less plaque buildup, and improved gingival response.

**Trial registration:**

The study protocol was retrospectively registered on Clinical Trials under No. (NCT06432049-29/05/2024).

## Background

The key of occlusion has been considered to be the first permanent molar (FPM). To preserve a healthy inter-occlusal relationship, FPM must be preserved [1]. The chewing ability of this side decreases by 40–45% because of its early loss [2]. Early FPM extraction has the following impacts, according to the chosen papers: cavities and/or fillings in neighbouring teeth; effects on incisors; effects on skeletal development; effects on post-extraction space; and effects on the development and eruption of the second permanent molar and third molar [3]. A recent study found that 35.16% of FPMs had a bad prognosis [4]. 8% of the 17-year-olds surveyed by another study conducted in the North of England had their FPMs extracted [5]. 

In children, the incidence of caries in FPMs is frequent. There are multiple reasons for it. First, the presence of deep pits and fissures in the occlusal surface morphology [6]. Second, the fact that most parents are unaware of it because of its early eruption date without a predecessor and the widespread misconception that the permanent teeth’s eruption sequence begins in the anterior area may be a contributing factor to its high caries incidence [7]. In addition, a primary molar that is carious may be next to it in the oral cavity due to its posterior position. Furthermore, the FPMs are vulnerable to a number of developmental abnormalities, such as hypoplasia and molar-incisor hypomineralization (MIH) [8]. Due to pain, more children are likely to seek restorations, root canal therapy, or even an early extraction of their first permanent molar [9]. 

Following endodontic therapy, a final restoration is required to maintain and safeguard the tooth’s remaining structure and to restore an acceptable function and appearance. The choice amongst a number of solutions is based on the tooth’s structural integrity, aesthetics, and need for protection [10]. 

It was proven to be challenging for all dentists to restore endodontically treated permanent molars in children and adolescents that are partially erupting due to caries, restoration failure, or developmental dental anomalies. These teeth exhibit weak biomechanical principles of retention and resistance due to diminished coronal structure, which is caused by caries and extensive cavity preparation, reducing the amount of remaining tooth structure [11]. In addition to inadequate interocclusal space caused by variations in crown length and height encountered during the transitional dentition [12]. 

SSCs are a practical restorative solution [13]. They are mostly used in deciduous teeth following pulp treatment. When it comes to permanent teeth, SSCs have the potential to function as an effective temporary treatment option for a fractured or damaged tooth until a permanent restoration can be completed. Additionally, they could serve as the restoration in a permanent molar that is partially erupted but needs full coverage [14]. SSC placement on permanent molars was found to be an extremely effective long-term interim repair that protects severely damaged adolescents molars until final prosthetic treatment is completed [15]. Although SSCs have been widely accepted for use on permanent molars, concerns have been expressed about their lack of accuracy in cervical fit, potential effects on periodontal tissues following placement, lack of aesthetics, challenges with tooth preparation, and SSCs adjustment [16]. With a follow-up of nearly four years, the overall success rate for SSCs was reported to be 88% in one retrospective clinical investigation. However, all unsuccessful SSCs were connected to periodontal problems [14]. 

Thus, esthetically pleasing, practical, and conservative substitutes are required. There are different resin adhesives with excellent bond strength and thin film thickness since adhesive dentistry has advanced significantly over the past few decades. High-strength restorative materials that can be etched and then luted with adhesives for improved retention are now available owing to the development of these materials. Endocrown is a clinically popular indirect restorative procedure that was established lately for the restoration of posterior teeth that have undergone endodontic treatment. Endocrown is a monoblock which is a single unit formed of monolithic ceramic crown that is anchored to the cavity borders and extends to the pulp chamber [17]. 

There has been little research on the use of endocrown restorations in children and their clinical outcomes, despite the literature’s discussion and illustrations of the restoration of first permanent molars with endocrowns and their survival in adults [18, 19]. Thus, the goal of this in vitro investigation was to assess the radiographic and clinical results of SSCs, computer-aided design-computer-aided manufacture (CAD-CAM) lithium disilicate glass-ceramic endocrowns, and CAD-CAM reinforced composite endocrowns. The null hypothesis will predict no difference in the clinical and radiographic results of the three restorative approaches.

## Materials and methods

A randomized clinical study was done in the department of pediatric dentistry and public health, faculty of dentistry, Delta University for Science and Technology. The study received approval from the oral and dental surgery faculty’s local ethical committee at Kafrelsheikh University, Kafrelsheikh, Egypt (MKSU/22-11-4) in the first of October 2022 and was carried out in compliance with the World Medical Association Declaration of Helsinki standards. This study was retrospectively recorded by clinical trials under No. (NCT06432049-29th of May 2024). The sample size was established using the formula for research involving purposive sampling, and the study lasted for a full year [20]. Seven children were needed for each group, assuming 80% power, 5% significance, and a 95% confidence interval. A total of ten children were included in each group for the possibility of dropouts.

Thus, this study consisted of 30 children (18 girls and 12 boys), with an age range of 10–13 years old, with an endodontically treated first permanent molar. Participants were chosen from those seeking dental treatment by the faculty members of the department of pediatric dentistry and public health (Delta University, Egypt). To assess the inclusion criteria, a clinical examination was part of the initial screening process.

**Fig. 1:**
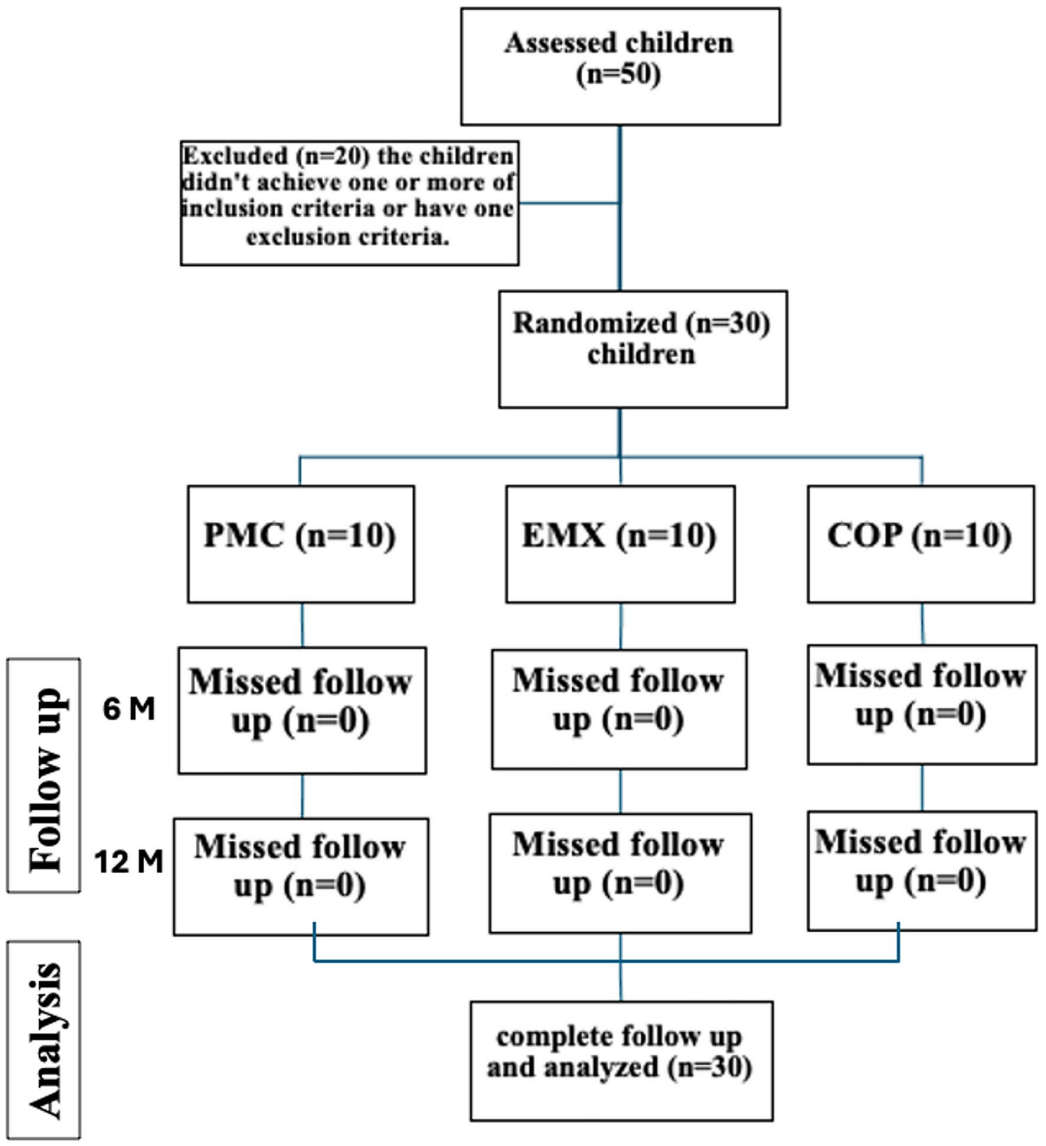
A flow chart for the present study

Thirty children were randomly distributed to three groups based on the type of the final restoration. All selected children were allocated to either PMC group (*n* = 10) restored with SSCs (Stainless-steel Molar Crowns, 3 M ESPE AG, Seafeld, Germany), EMX group (*n* = 10) endocrown fabricated from CAD-CAM lithium disilicate glass ceramic (e.max CAD, Ivoclar AG, Schaan, Liechtenstein), or COP group (*n* = 10) endocrown fabricated from CAD-CAM reinforced composite (Brilliant Crios, Coltene, Altstätten, Switzerland) as illustrated in flow chart Fig. 1.

The patients were chosen based on the subsequent inclusion criteria:

1. Patient with mature first permanent molar and with an age range of 10 to 13 years old.
2. Occlusion that is normal and devoid of parafunctional behaviours that were assessed clinically or through patient history.
3. Mandibular molar requiring root canal treatment.
4. After occlusal reduction for endocrown, a supra-gingival margin was needed for rubber dam application.
5. Lack of cracks or fractures in the roots.

While the exclusion criteria:

1. Difficulty of rubber dam application which, is mandatory for adequate endocrown bonding.
2. Inability of the patient to cooperate with follow-up and post-operative recall.
3. A high caries index and poor dental care.

Each patient’s parent was informed about the treatment in their own language, and a signed informed consent form was acquired. Subsequently, root canal treatment was started using a diamond bur mounted on a high-speed handpiece with copious water spray to access the pulp chamber. After that, root canals were prepared using full-sequence rotary files (Protaper Gold, Dentsply Sirona, Ballaigues, Switzerland).

3% NaOCl, 17% ethylene diamine tetraacetic acid, and 0.9% normal saline were used for irrigation [21]. After taking a master cone radiograph, every canal was sealed using a bioceramic sealer and obturated using a single cone obturation procedure. After applying a temporary restoration (CavitG, 3 M ESPE Dental-Medizin GmbH Co., Seafeld, Germany), an intra-oral periapical radiograph was taken postobturation. The temporary filling was left for not more than seven days before starting the final restoration procedures.

Using randomised trial software (Researcher Randomiser), patients were randomly assigned to three groups one week following endodontic treatment. To hide the allocation, a researcher who was not involved in the therapy or result assessment carried out the random allocation.

For group PMC, teeth were prepared to receive SSCs with the following procedures:

- Occlusal reduction was carried out to obtain clearance of approximately 1.5 mm. The mesial and distal contact points were cleared, and a smooth taper from occlusal to gingival was obtained that is free of ledges or shoulders.
- All sharp line angles were rounded off. The finish line was placed approximately 1 mm below the gingival margin.
- Using callipers, the mesio-distal width between the contact points of the neighboring teeth was measured in order to choose the appropriate size crown.
- The mesio-distal width of the corresponding tooth on the opposing side can be used if the neighbouring tooth was absent.
- Traditional glass ionomer (GC Fuji I, GC CO., Tokyo, Japan) was used to cement the crown.
- The crown is positioned lingually and rolled over the preparation to the buccal edge in order to seat it on a prepared tooth.
- A crown’s audible “click” sound occurs when it covers the gingival undercut region. Usually, the crown needs to be seated firmly. With a well-fitting crown, the marginal gingiva will slightly blanch as it seats. Approximately 1 mm subgingivally is where the crown edge should be placed for both retention and a strong cement seal.

**Fig. 2:**
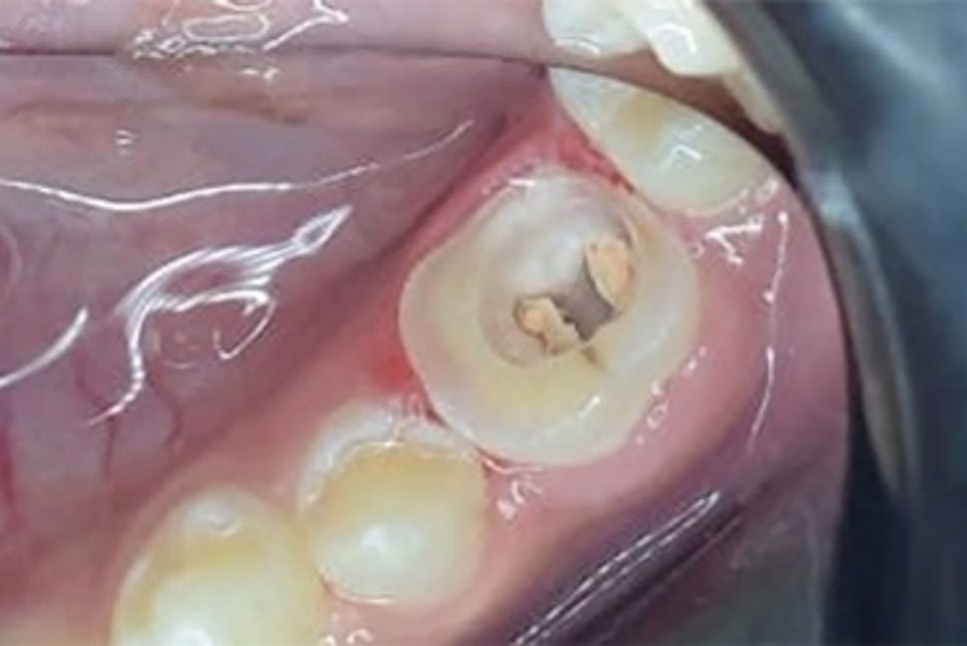
An example of endocrown preparation

Regarding the endocrown groups (group EMX and group COP), the preparation was done as shown in Fig. 2 with the following procedures:

- Cusps were reduced to produce a butt joint margin with minimal 2 mm thickness with a flat diamond bur (TF-13-No.173/019, Mani, Utsunomiya, Japan).
- A weel-shaped diamond bur (WR-13 C-No.198/018, Mani, Utsunomiya, Japan) was used to ensure a smooth, flat butt joint.
- The wall of the pulp chamber was prepared with a tapered diamond rotary instrument with rounded end (TR-13-No.198/018, Mani, Utsunomiya, Japan) held perpendicular to the floor of the pulp chamber. A total occlusal convergence of (7º to 10º) was prepared.
- All line angles of the pulp chamber were rounded and smoothed.
- The floor of the pulp chamber was filled with flowable resin composite (Tetric N-flow, Ivoclar AG, Schaan, Liechtenstein) to provide a smooth, flat surface.
- The butt joint and walls of the pulp chamber were finally finished.

- After preparations, impressions were taken using a one-step technique with additional silicon material (Elite HD+, Zermackh, Badia Polesine, Italy).
- Wax bite registration was taken for each child, and both were forwarded to the technician.
- Following, the impressions were poured and scanned in the laboratory. The standard tessellation language (STL) file, which was a virtual image of the model, was exported and transferred to a software program (DentalCAD 3.0 Galway 2021, exocad, Darmstadt, Germany) that was used to design the endocrown restoration.
- After the endocrown was designed to provide correct occlusion and a cement space of 40 μm, the STL file was transferred to a 5-axis milling machine (Coritec 250i, imes-icore GmbH, Eiterfeld, Germany).
- The CAD-CAM material that was chosen for every group was used to mill the endocrown restorations. The remaining sprue was finished with a diamond wheel (DCB, Schleifer, Komet Dental, Lemgo, Germany) following milling.
- Endocrowns for group EMX were placed on a crystallisation tray and crystallised in a furnace (Vita Vacumat 6000 M; VITA Zahnfabrik GmbH, Bad Säckingen, Germany).
- Following milling, the endocrown restorations were assessed in the patient’s mouth, and pressure sites were marked utilising a water-soluble pressure-indicating paint (PIP; Keystone Industries, Singen, Germany).
- All pressure areas found were eliminated using a finishing green diamond tip (DCB, Schleifer, Komet Dental, Lemgo, Germany) until full seating was confirmed. The same occlusion was verified with articulating paper (straight blue-red, thin, Produits Dentaires SA, Vevey, Switzerland) in both centric and eccentric contact, and any high contact was removed until the correct occlusion was maintained.
- After try-in of endocrown, the tooth was isolated with a rubber dam to ensure good adhesion.

Cementation of endocrown

**Fig. 3:**
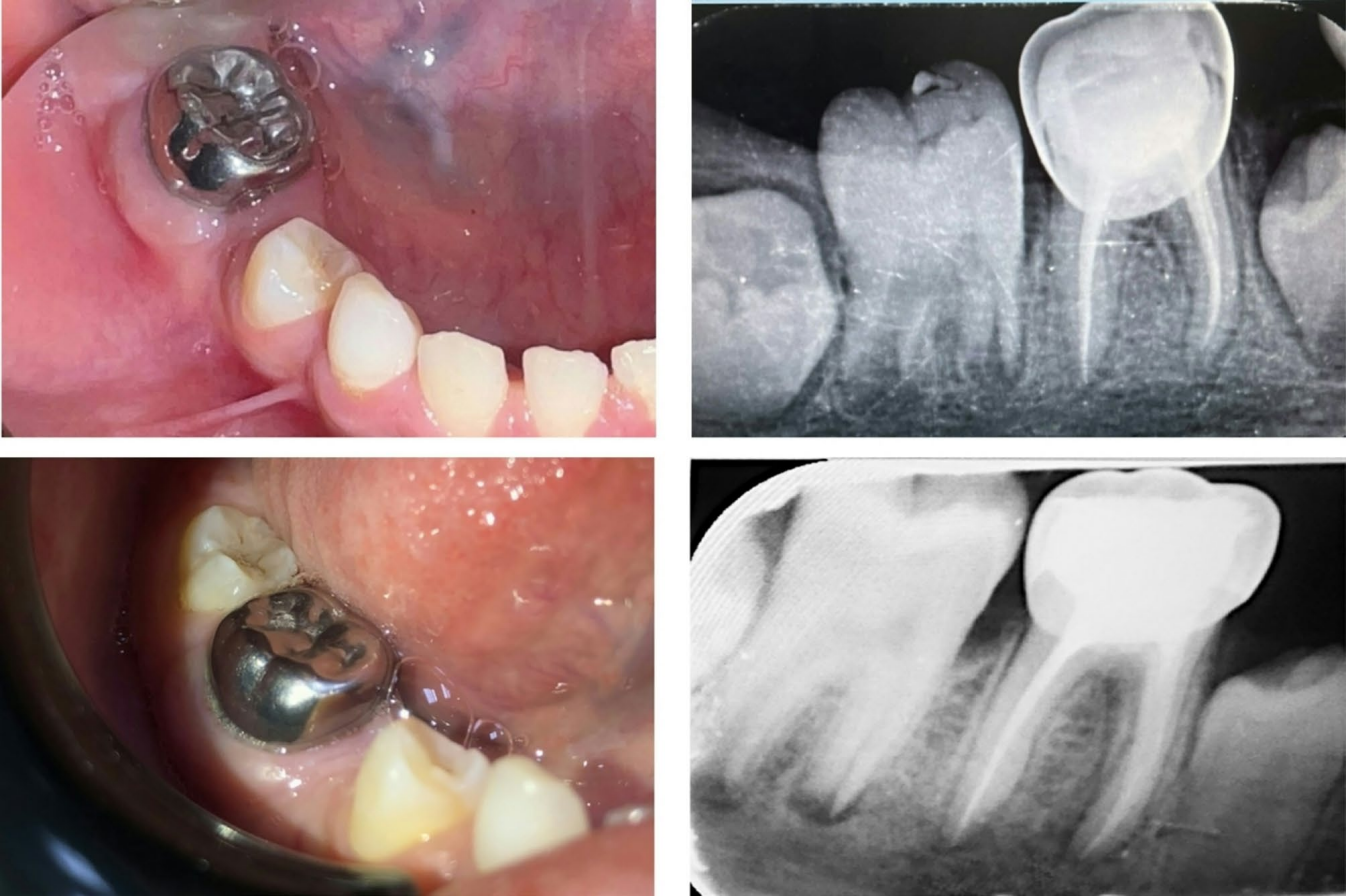
**A-d** An example of clinical picture and radiograph for PMC group at base line and after 12 months follow up

**Fig. 4:**
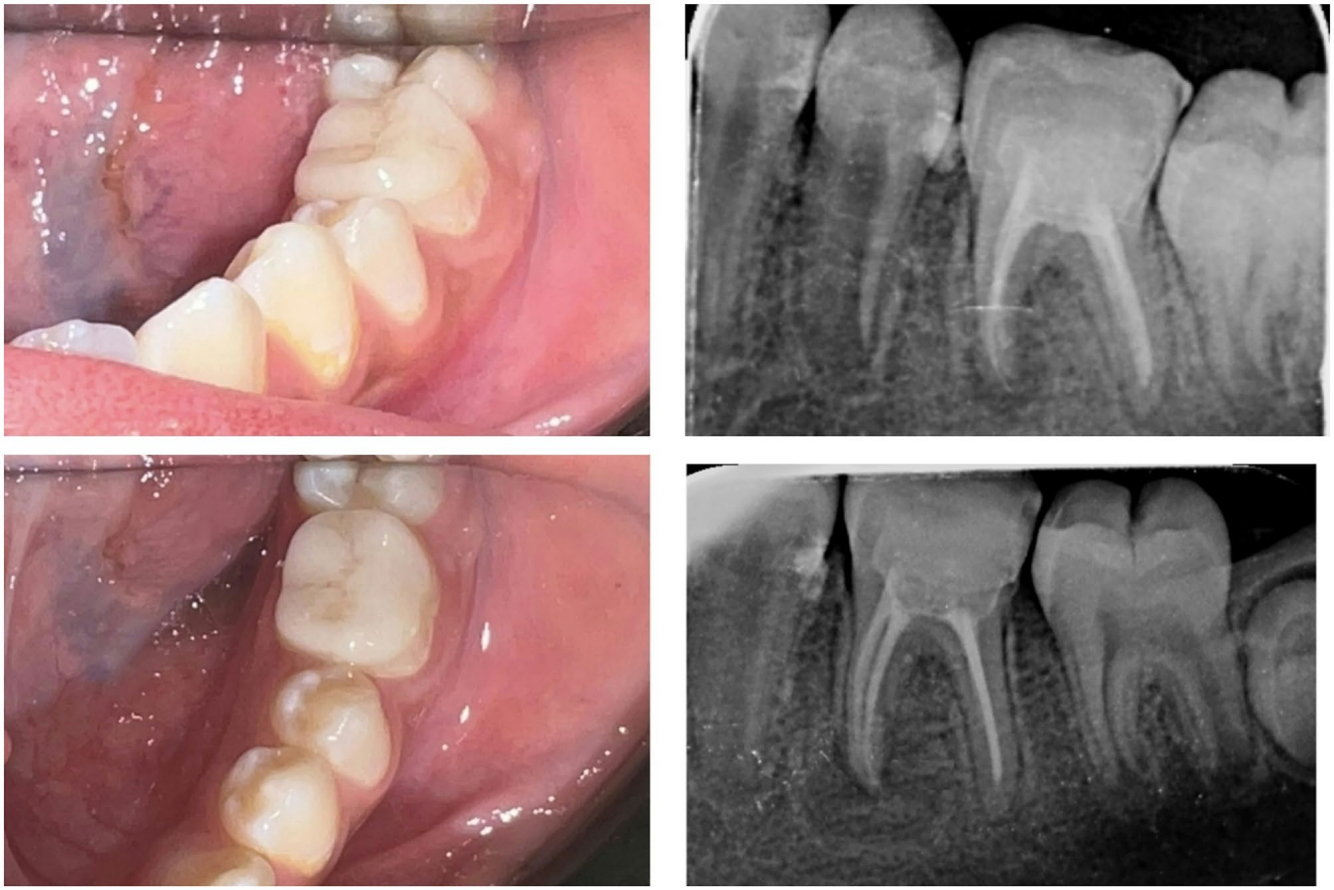
**A-d** An example of clinical picture and radiograph for EMX group at base line and after 12 months follow up

**Fig. 5:**
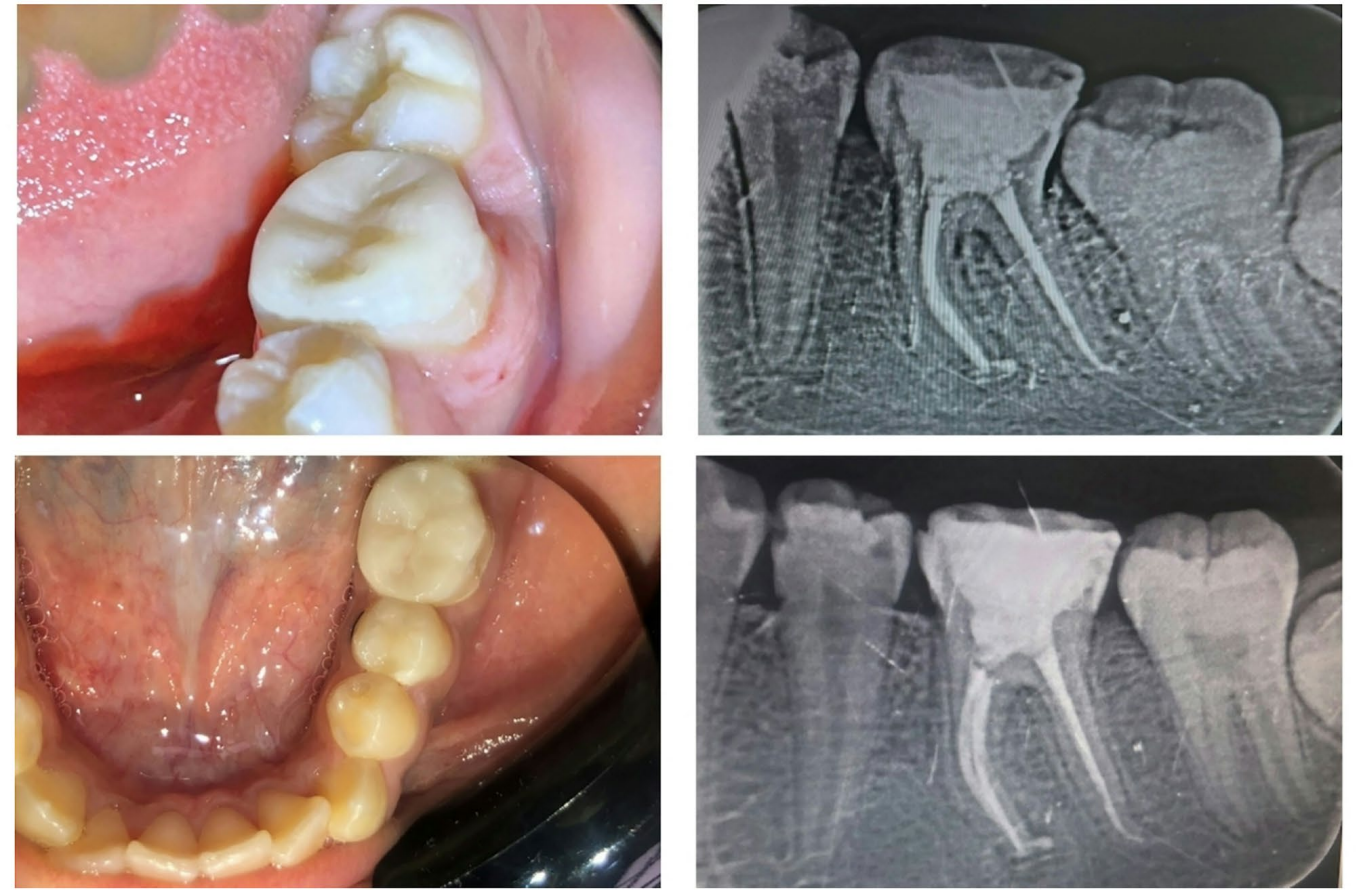
**A-d** An example of clinical picture and radiograph for COP group at base line and after 12 months follow up

- Different protocols of surface treatment were used according to the material of the endocrown. For the EMX group, hydrofluoric acid etch gel 4.5% (Porcelain etch, Ultradent Products, Cologne, Germany) was applied to the fitting surface for 20 s, rinsed, and dried, followed by application of silane coupling agent.
- For the COP group, the fitting surface was sandblasted with 50 μm aluminum oxide (Al2O3) particles from a working distance of 10 mm for 10 s, followed by surface cleaning with phosphoric acid etchant gel (37%). Finally, a universal adhesive (one coat 7, Coltene, Altstätten, Switzerland) was applied and air dried.
- The enamel surface of the tooth was treated for 30 s with an Ultra-Etch (Ultra-Dent Products, Cologne, Germany) etch containing 37.5% phosphoric acid, rinsed, and dried.
- For endocrowns cementation, a self-adhesive resin cement (Rely-X Unicem, 3 M ESPE, St. Paul, USA) was used, while a constant, hard pressure was digitally applied and stabilised. The margins of endocrowns were treated for 2 s with LED-based visible light cure (Bluephase G2, Ivoclar AG Schaan, Liechtenstein). After removing any leftover cement, each surface was given a further 20 s of light curing.
- Following cementation, all children were evaluated over one year at baseline, 6 months, and 12 months, as shown in (Fig. 3A-D), (Fig. 4A-D), and (Fig. 5A-D) for the following criteria:
- Radiographic evaluation of tooth for the evidence of periapical pathology or recurrent caries at the margin of restorations.
- Parent satisfaction was measured by using a 1 to 5 Likert-type scale [22], with 1 being very dissatisfied, 2 being dissatisfied, 3 being fair, 4 being satisfied, and 5 being very satisfied, in terms of shade, durability, size, and overall esthetics.
- Plaque index (PI) was evaluated as described by Silness and Löe [23].

0 = No plaque.

1 = a layer of plaque that is invisible to the naked eye that covers the tooth’s free gingival edge and neighboring teeth. However, only with the use of a probe or disclosing solution.

2 = Moderate collection of deposits that are visible to the naked eye in the gingival pocket, on the gingival margin, and/or on the surface of the neighboring tooth.

3 = Soft matter is abundant on the tooth and gingival border, as well as in the gingival pocket.

- Gingival index (GI) was evaluated according to the scoring system developed by Löe and Silness [24].

0 = Normal gingiva.

1 = Mild inflammation, including a little colour change and mild oedema. No bleeding while probing.

2 = Redness, oedema, and glazing are signs of moderate inflammation. Bleeding when probed.

3 = severe inflammation, including ulceration and noticeable redness and oedema. potential for continuous bleeding.

The operators who administered the treatment will not serve as the evaluators in order to avoid information bias.

### Statistical analysis

Data were entered and analysed using IBM-SPSS software (IBM Corp. released 2020. IBM SPSS Statistics for Windows, Version 27.0. Armonk, NY: IBM Corp). The median (lowest-maximum) and N (%) were used to express ordinal data. An ordinal variable recorded at three different levels within a group was compared using Friedman’s test. Pairwise comparisons were provided for each difference that was statistically significant utilising the Bonferroni adjustment for multiple comparisons. To compare an ordinal variable assessed between the three groups, the Kruskal-Wallis H-test was employed. Kendalls uses Cohen’s interpretation guidelines of 0.1 - < 0.3 (small effect), 0.3 - < 0.5 (moderate effect), and > = 0.5 (large effect). The interpretation values commonly in published literature are: 0.01- < 0.06 (small effect), 0.06 - < 0.14 (moderate effect), and > = 0.14 (large effect). If *P* ≤.050 for any of the tests used, the results were deemed statistically significant. Where appropriate, charts were utilised to visually represent the results.

## Results

**Table 1:** Baseline demographic parameters

Parameter at baseline	PMC	EMX	COP	*P* value
Age (years)	11.8 ± 0.79	11.9 ± 0.74	12.1 ± 0.74	0.668
Gender				
Male	4 (40%)	3 (30%)	5 (50%)	0.893
Female	6 (60%)	7 (70%)	5 (50%)	

Notes: Age data: means ± SD (= standard deviation), while gender data: N (%). The test of significance is one-way ANOVA for age and Fisher-Freeman-Halton Exact Test for gender

Table 1 showed no statistically significant differences in age and gender between the three groups.

## Radiographic evaluation

The radiographs showed no evidence of pathological problems such as periapical lesions or recurrent caries at margins of restorations in all groups through the 12-month followup. The radiographs showed no radiolucency at the periapical or beneath the margins of restorations. Clinically, no cases showed margin discoloration, chipping, fracture, or debonding of restoration.

## Parent satisfaction

**Table 2:** The descriptive statistics and Kruskal-Wallis test for parent satisfaction as shown in Fig. 6

Group	No.	Parent satisfaction score			H [2]	η^2^ [H]	*P*-value
		Median	Minimum (N%)	Maximum (N%)			
PMC	10	3	3 (60%)	5 (20%)	20.714	0.693	**<.001**
EMX	10	5	5 (100%)	5 (100%)			
COP	10	5	5 (100%)	5 (100%)			

Notes: H [2] = H-statistic at 2 degrees of freedom. η^2^ [H] = eta squared based on H = statistic = a measure of effect size. The test of significance is Kruskal-Wallis H-test

Kruskal-Wallis H-test

Table 2 showed that the parent satisfaction scores were statistically significantly different between the three groups, H [2] = 20.714, *P* <.001.

A post hoc analysis for a pairwise comparison revealed statistically significant differences in parent’s satisfaction scores between PMC (mean rank = 7.5) vs. both EMX (mean rank = 19.5) (*P* <.001) and COP (mean rank = 19.5) (*P* <.001), but not between the EMX and COP groups (*P* = 1.00).

**Fig. 6:**
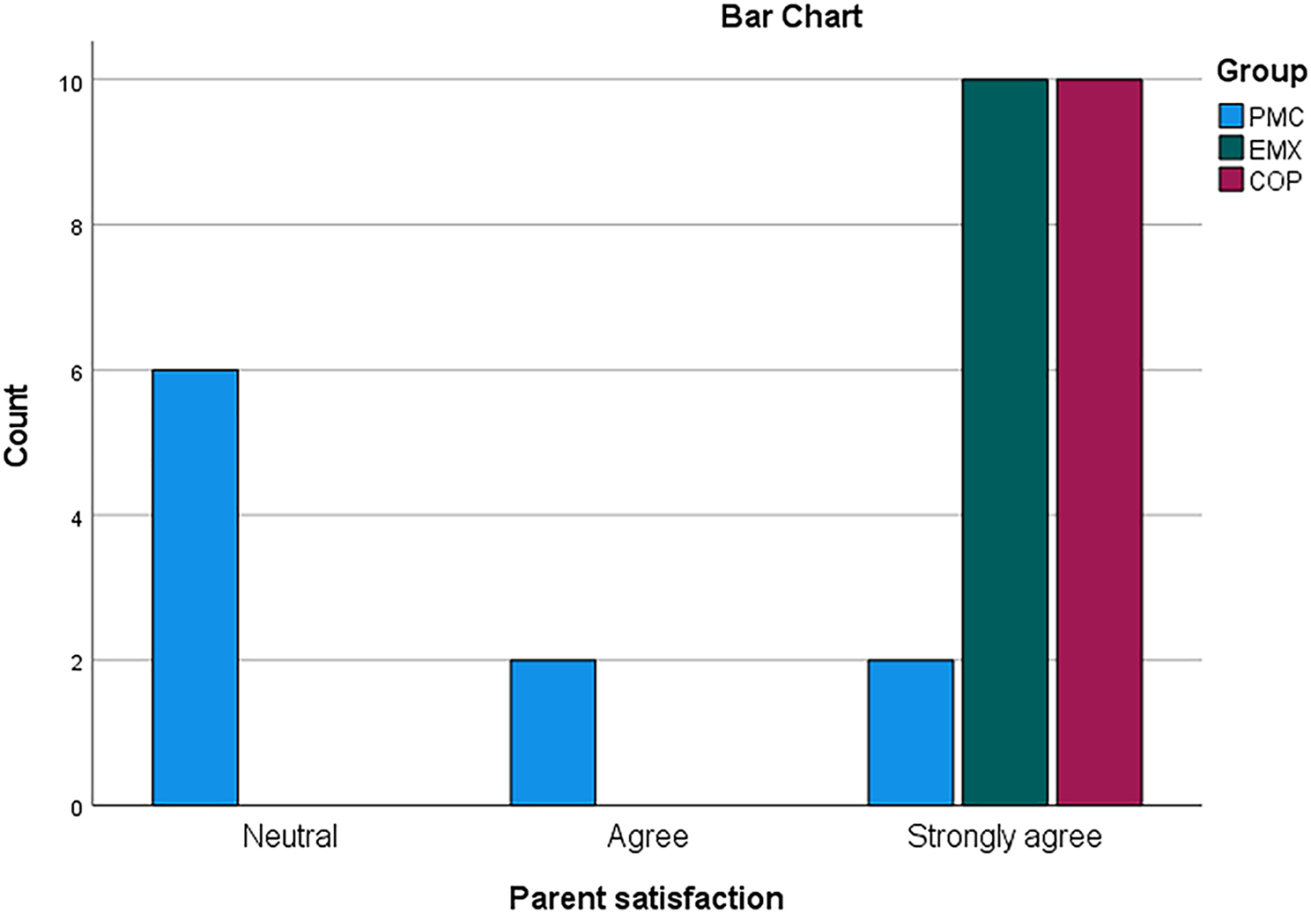
The parent satisfaction variation and count in 3 compared groups

## Plaque index

**Table 3:** Related-Samples Friedman’s Two-Way Analysis of Variance by Ranks Summary in three groups for PI as shown in Fig. 7

**Group**	**No.**	**Gingival index**												
		**Base line**			**6- months**			**12- months**				**χ** ^**2**^ **[2]**	***Kendall’s W***	***P*** **-value**
		**Median**	**Minimum**	**Maximum**	**Median**	**Minimum**	**Maximum**	**Median**	**Minimum**		**Maximum**			
**PMC**	**10**	**0**	**0**	**0**	**1**	**1**	**1**	**1**	**1**		**1**	**20.000**	**1.333**	**< 0.001**
**EMX**	**10**	**0**	**0**	**0**	**0**	**0**	**1**	**0**	**0**		**0**	**4.000** ^**a**^	0.267	0.135
**COP**	**10**	**0**	**0**	**0**	**0**	**0**	**1**	**0**	**0**		**1**	**4.000** ^**a**^	0.267	0.135

Notes: Data is median (minimum– maximum). The test of significance is Friedman’s test. Significance values have been adjusted by the Bonferroni correction for multiple tests

Table 3 showed that there were significant changes in PI values in PMC group (*P* <.000) between base line (0) and 6-months (1) and changes remained the same at 12 months, while there were no significant changes in both EMX and COP groups through different intervals with the same P-value (*P* =.135).

**Fig. 7:**
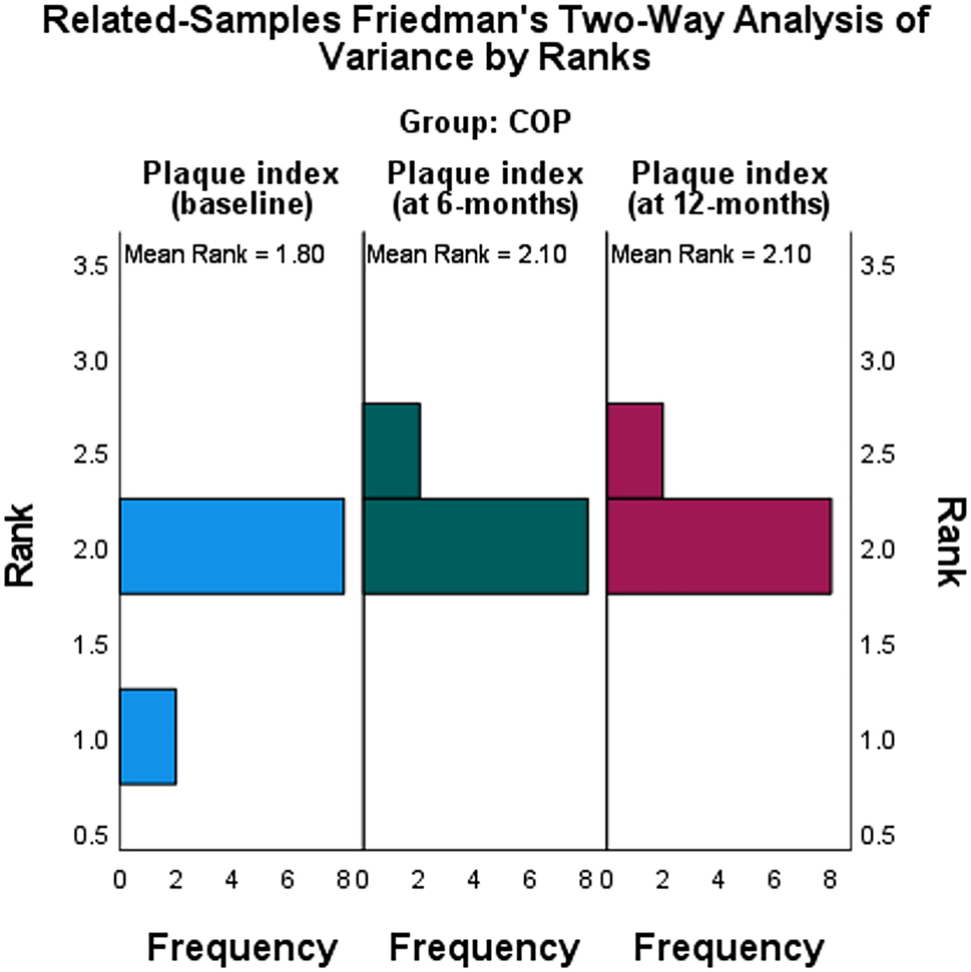
The PI frequency at different intervals in PMC group

**Table 4:** Pairwise comparison of PI in PMC group

Group	Base line vs. 6- months	Base line vs. 12- months	6 months vs. 12-months
PMC	**0.002**	**0.002**	**1.000**

Table 4 showed a pairwise comparison of PI values between intervals in the PMC group. It was found that there was a significant difference between PI values in the baseline and both 6-month and 12-month follow-ups (*P* =.002). While there was no significant difference in PI values between 6-month and 12-month follow-ups (*P* = 1.000).

**Table 5:** Comparisons of PI between groups at each time point

Time	PMC		EMX		COP		H [2]	η^2^ [H]	*P*
	Median	Min-Max	Median	Min-Max	Median	Min-Max			
Baseline	0	0–0	0	0–0	0	0–0	0.000	0.074	1.00
6-months	1	1–1	0	0–1	0	0–1	16.57	0.540	< 0.001
12-months	1	1–1	0	0–0	0	0–1	22.55	0.761	< 0.001

Notes: Min-Max = Minimum-Maximum. H [2] = H-statistic at 2 degrees of freedom. Sig. = *P*-value. The test of significance is Kruskal-Wallis H-test

In comparison of PI values between the 3 groups at different intervals, as shown in Table (5), it was found a statistically significant difference between the 3 groups at 6-months and 12-months (*P* <.000). While there was no significant difference in PI values at the base line.

**Table 6:** Pairwise comparisons of PI between 3 groups at 6-months

Interval	PMC vs. EMX	PMC vs. COP	EMX vs. COP
6-months	0.001	0.001	1.000
12-months	< 0.001	0.001	1.000

Table 6 showed a pairwise comparisons of PI values between 3 groups revealed that PI values of the PMC group were statistically significantly higher than those of both the EMX and COP groups at 6-months (*P* =.001) and 12-months (*P* <.001 and *P* =.001 respectively).

### Gingival index

**Table 7:** Related-Samples Friedman’s Two-Way Analysis of Variance by Ranks Summary in three groups for GI as shown in Fig. 8

Group	No.	Gingival index											
		Base line			6- months			12- months			χ^2^ [2]	Kendall’s W	*P*-value
		Median	Minimum	Maximum	Median	Minimum	Maximum	Median	Minimum	Maximum			
**PMC**	**10**	**0**	**0**	**0**	**0**	**0**	**1**	**0**	**0**	**1**	**6.000**	**0.400**	**0.050**
**EMX**	**10**	**0**	**0**	**0**	**0**	**0**	**0**	**0**	**0**	**0**	**0.000** ^**a**^	**0.000**	**1.000**
**COP**	**10**	**0**	**0**	**0**	**0**	**0**	**1**	**0**	**0**	**1**	**4.000** ^**a**^	**0.267**	**0.135**

Notes: Data is median (minimum– maximum). The test of significance is Friedman’s test. Significance values have been adjusted by the Bonferroni correction for multiple tests

Table 7 showed that there were changes in GI values in the PMC group with statistically significant difference through intervals (*P* =.050), while there was no significant changes in both the EMX and COP groups through different intervals with *P*-values (*P* = 1.000) and (*P* =.135) respectively.

**Fig. 8:**
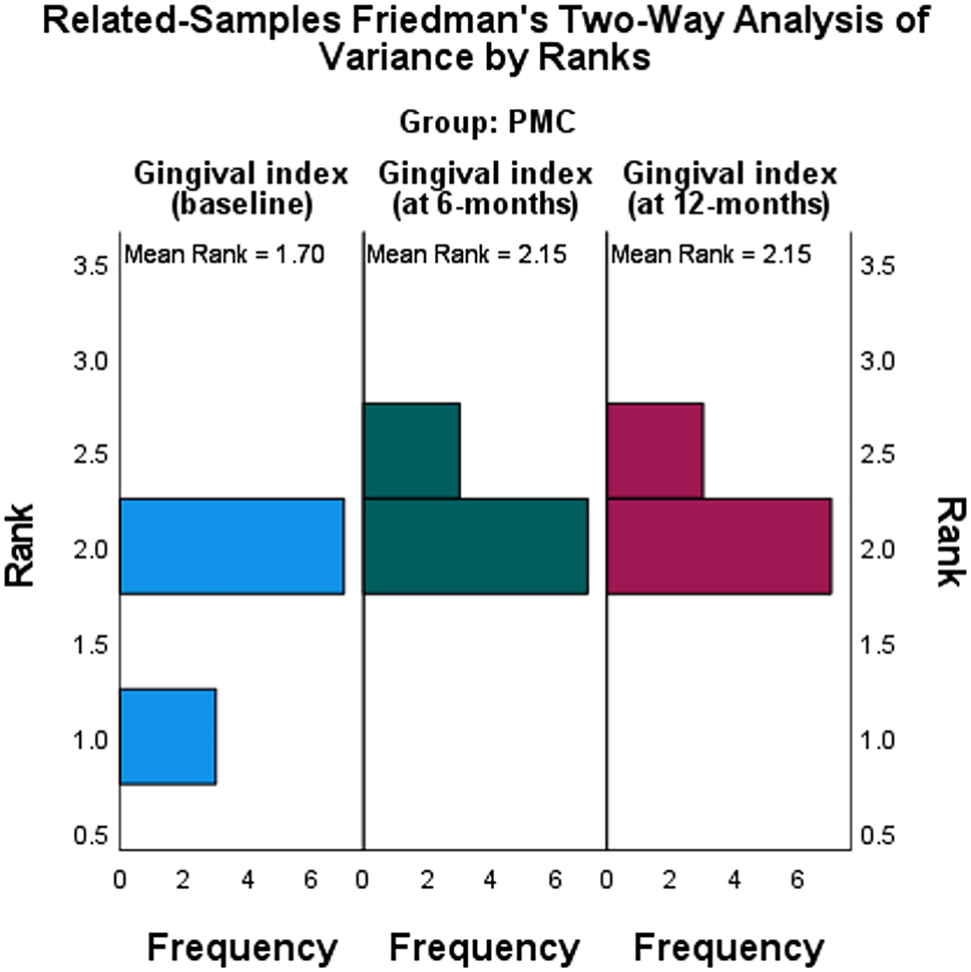
The GI frequency at different intervals in PMC group

**Table 8:** Pairwise comparison of GI in PMC group

Group	Base line vs. 6- months	Base line vs. 12- months	6 months vs. 12-months
PMC	0.943	0.943	1.000

While in a pairwise comparison of GI values between intervals in the PMC group as shown in Table 8, it was found that there was no significant difference between GI values in the baseline and both 6-month and 12-month follow-ups (*P* =.943), nor between GI values between 6-month and 12-month follow-ups (*P* = 1.000).

**Table 9:** Comparisons of GI between groups at each time point

Time	PMC		EMX		COP		H [2]	η^2^ [H]	*P*-value
	Median	Min-Max	Median	Min-Max	Median	Min-Max			
Baseline	0	0–0	0	0–0	0	0–0	0.000	0.074	1.00
6-months	0	0–1	0	0–0	0	0–1	3.248	0.046	0.197
12-months	0	0–1	0	0–0	0	0–1	3.248	0.046	0.197

Notes: Min-Max = Minimum-Maximum. H [2] = H-statistic at 2 degrees of freedom. Sig. = *P*-value. The test of significance is Kruskal-Wallis H-test

In comparison of GI values between the 3 groups at different intervals, as shown in Table 9, it was found that there was no significant difference.

## Discussion

The results of the present clinical study showed that all cases in 3 groups survived through the 12 months of the study without any complications of fracture or debonding. The study’s null hypothesis was partially rejected because there were significant differences between the endocrown groups and the stainless steel group in parent satisfaction and PI values, but the GI values showed no significant difference.

It is well recognised that dentists can be inconsistent when determining the optimum way to restore teeth that have had endodontic therapy, particularly the permanent teeth in younge children [17, 25]. Previous findings indicated that full cusp coverage is necessary to safeguard endodontically treated posterior teeth, as this will impact long-term outcomes [25]. The coronal microleakage and bacterial contamination that happen when endodontically treated teeth are not promptly restored, leading to endodontic failure and necessitating retreatment, is another problem associated with such teeth [26]. 

For nearly fifty years, SSCs were a good option for transitional restorations of permanent molars in children. This findings indicated that SSCs are valued for durability and cost-effectiveness [27]. However, there were several drawbacks to using SSCs, such as their pre-sized size, poor marginal adaptation, high risk of recurrent caries, periodontal inflammation, and impaction of erupting teeth [14, 28, 29]. 

Because of its supragingival borders, endocrown restorations need less tooth reduction than SSCs, do not require post and core restorations, safeguard tooth structure, offer excellent strength for cuspal overlays, and preserve periodontal health [11, 30]. The patient’s short- and long-term demands, the child’s and parents’ participation, the expense of the procedure, the clinician’s experience, and the materials available should all be taken into consideration when deciding whether to repair endodontically treated molars using indirect adhesive restorations or prefabricated SSCs [31]. 

Endocrowns are a more straightforward treatment with a shorter chair time, less preparation processes, and fewer challenges as compared to a typical post and core system than other restorative choices [32]. Numerous studies have documented the issues with post-space preparation, including the potential for root fracture and over-weakening of the tooth due to radicular dentin removal [25]. 

With the adhesive technology provided by the endocrown restoration, paediatric dentists can restore teeth that have undergone endodontic treatment with CAD-CAM components in a conservative and successful manner [33–35]. Children’s teeth can be restored with endocrowns, which have achieved clinical recognition for the restoration of endodontically treated adult teeth [34]. 

When compared to traditional crowns, the tensile strength of endocrowns is expected to be higher because of the increased coronal thickness and covering of the pulp extensions and margins, which subject the ceramic to compressive load [34, 36]. Furthermore, compared to conventional crowns supported on fibre posts, Biacchi and Basting reported that lithium disilicate endocrowns exhibited a higher resistance to compressive force [37]. Finite element research has highlighted the significance of endocrowns in stress distribution lately [38]. 

Endocrowns performance was as good as other restorative treatments or even better, according to a comprehensive study that compared their clinical outcome and fracture resistance (in vitro) to direct composite, inlay/onlay indirect restorations, and intraradicular posts [35]. 

Clinical Outcomes.

The most prominent result of the present study was for parent satisfaction, as it has been noticed that esthetic endocrowns were more satisfying to parent than stainless steel crowns, with a significant difference (*P* <.001). Although endocrown is an indirect restoration and needs more visits, parents were satisfied because of its satisfying colour. This finding might be due to the availability of choosing the shade of the endocrown restoration. In addition, it was a final restoration, with less complex procedures, more practical, and less chair time [11]. 

According to the author’s knowledge, until now, there was no literature on parent satisfaction for endocrown in the FPM. When comparing parent satisfaction with endocrowns in primary molars to zirconia crowns, there was no statistically significant difference, according to a study by Khattab et al. [39] However, there was a significant difference in the parent satisfaction with colour because the colour of the endocrown can be chosen to mimic the structure of the tooth, whereas the colour selection for the zirconia crown was restricted.

Regarding plaque index, the PMC group showed significant changes from 0 to 1 after six months and continued the same after 12 months (*P* <.001), although all children were instructed regularly to practice oral hygiene, while COP and EMX showed insignificant changes through different intervals. Most children in the COP and EMX groups showed no plaque in the area adjacent to the gingiva, which is significantly different from that of the PMC group at 6-months and 12-months (*P* <.001), which showed a plaque in the form of a thin film on the gingival margin. While there was no significant difference in PI values at the baseline between different groups. Similar findings were reported by Heidari et al., [40] who demonstrated that, as long as the patient practices appropriate oral hygiene, properly fitted SSCs have no negative effects on the gingiva of permanent first molars. On the other hand, GI and PI significantly increased statistically in permanent molars replaced with stainless steel crowns from baseline to follow-up, according to another research investigation by Koleventi et al. [13] Numerous factors, including the patient’s hygiene practices, bacterial flora, and periodontal maintenance, may contribute to plaque accumulation [41]. 

The most noticed results of GI showed healthy gums for the EMX group through different intervals with no changes. While PMC and COP groups showed insignificant changes in GI values that represented mild inflamation with no bleed in probing in some cases, that did not have a negative impact on the survival of restoration. In comparisons of GI values between groups through different intervals, there was no significant difference. There were no notable difference in GI values between groups despite variations in PI, which might be because follow-up visits included repeated oral hygiene education. It was found that frequent, efficient oral hygiene practices might reduce the amount of GI values [42]. Insignificant changes in GI for endocrown over time may be caused by the restorations’ supragingival margin, which prevents gingiva breach during tooth preparation, was linked to the gingival health in the endocrown groups. Additionally, lithium disilicate affords high levels of biocompatibility due to low plaque retention, which has been approved in previous research [43]. Ibrahem et al. [44] reported similar findings, concluding that there were no negative impacts of endocrowns on periodontal health as observed clinically.

Although the findings of current research favor the use of endocrown for FPM in children, some clinical situation will indicate the use of SSCs. When teeth exhibit moderate to severe hypoplasia and following root canal therapy, SSCs are the recommended course of action [17, 45]. Full-coverage restorations using SSCs are preferred over endocrown restorations due to a number of factors, including the prevention of further tooth deterioration, the development of appropriate occlusal relationships and interproximal contacts, the relatively low technique sensitivity and costs, and the quick preparation and insertion times [11, 32]. Another challenge for clinical application of endocrown is the cost of CAD-CAM systems that can not be afforded by patients.

## Limitations and future directions

The two limitations of the current in vivo investigation were its limited sample size and short follow-up period. The incresed sample size may provide better generalizability and the longer follow up period may affect survival rates or periodontal health outcomes. Thus, it is recommended to make future studies with larger sample size and long period of follow up. Additionaly, it is recommended to use digital impression for easier and more precise procedures.

## Conclusions

Within limitation of this study.

- Both SSCs and endorowns showed promising survival rate.
- Endocrowns showed higher parent satisfaction than SSCs with statistical significance because of esthetics and it was a premanent restoration.
- Endocrowns showed less plaque accumulation than SSCs with statistical significance, which resulted in better gingival and plaque indicies.
- Future studies investigating long-term outcomes and clinical performance of endocrowns with a larger sample size are recommended.
- Permanent endocrown restoring FPM in children will decrease future interventions, thus decreasing dental anxiety.

## Data Availability

The datasets generated and/or analyses during the current study are not publicly available due to [the research is not published yet] but are available from the corresponding author on reasonable request.

## Abbreviations

FPM: First permanent molar
SSCs: Stainless-steel crowns
CONSORT: Consolidated Standards of Reporting Trials GI Gingival index
PI: Plaque index
GI: Ginigval index
CAD-CAM: Computer aided design - computer aided manufacture
IRC: Indirect Resin Composite
STL: Standard Tessellation Language
